# Abnormal Brain Network Topology During Non–rapid Eye Movement Sleep and Its Correlation With Cognitive Behavioral Abnormalities in Narcolepsy Type 1

**DOI:** 10.3389/fneur.2020.617827

**Published:** 2021-01-11

**Authors:** Xiaoyu Zhu, Kunlin Ni, Huiwen Tan, Yishu Liu, Yin Zeng, Bing Yu, Qiyong Guo, Li Xiao

**Affiliations:** ^1^Department of Radiology, Shengjing Hospital of China Medical University, Shenyang, China; ^2^Department of Pulmonary and Critical Care Medicine, Shengjing Hospital of China Medical University, Shenyang, China; ^3^Sleep Medicine Center, Shengjing Hospital of China Medical University, Shenyang, China

**Keywords:** cognitive dysfunction, graph theory analysis, type 1 narcolepsy disorder, functional connectivity, sleep, Montreal cognitive assessment Beijing edition

## Abstract

**Objective:** Simultaneous electroencephalography (EEG) and functional magnetic resonance imaging (fMRI) were applied to investigate the abnormalities in the topological characteristics of functional brain networks during non-rapid eye movement(NREM)sleep. And we investigated its relationship with cognitive abnormalities in patients with narcolepsy type 1 (NT1) disorder in the current study.

**Methods:** The Beijing version of the Montreal Cognitive Assessment (MoCA-BJ) and EEG-fMRI were applied in 25 patients with NT1 and 25 age-matched healthy controls. All subjects participated in a nocturnal video polysomnography(PSG)study, and total sleep time (TST), percentage of TST (%TST) for each sleep stage and arousal index were calculated. The Epworth Sleepiness Score (ESS) was used to measure the degree of daytime sleepiness. The EEG-fMRI study was performed simultaneously using a 3T MRI system and a 32-channel MRI-compatible EEG system during sleep. Visual scoring of EEG data was used for sleep staging. Cognitive function was assessed for all subjects using the MoCA-BJ. The fMRI data were applied to establish a whole-brain functional connectivity network for all subjects, and the topological characteristics of the whole-brain functional network were analyzed using a graph-theoretic approach. The topological parameters were compared between groups. Lastly, the correlation between topological parameters and the assessment scale using Montreal Cognition was analyzed.

**Results:** The MoCA-BJ scores were lower in patients with NT1 than in normal controls. Whole-brain global efficiency during stage N2 sleep in patients with NT1 displayed significantly lower small-world properties than in normal controls. Whole-brain functional network global efficiency in patients with NT1 was significantly correlated with MoCA-BJ scores.

**Conclusion:** The global efficiency of the functional brain network during stage N2 sleep in patients with NT1 and the correspondingly reduced small-world attributes were associated with cognitive impairment.

## Introduction

NT1 is a chronic neurological disorder characterized by irresistible daytime sleepiness, cataplexy, sleep hallucinations, sleep paralysis, and nocturnal sleep disturbances (1). Patients with NT1 typically show cataplexy and a dramatic reduction in the cerebrospinal fluid concentration of the neuropeptide orexin A (hypocretin 1), which results from extensive loss of the orexin-producing neurons in the hypothalamus (2).

Several current psychiatric studies on patients with NT1 disorder have suggested that these patients have multiple cognitive deficits, including abnormal emotional learning (3), deficits in subjective perception of attention (4), lack of perseverance and selectivity in decision making (5), and deficits in the ability to activate attention and arousal-related areas (6).

To explore the neural mechanisms underlying NT1 disorder combined with cognitive impairment, several neuroimaging studies have been conducted in the last decade. Among them, structural imaging studies have revealed significant abnormalities in the bilateral hippocampus, amygdala (7–10) and left marginal superior gyrus (11) gray matter, as well as in the bilateral cerebellar hemispheres, bilateral thalamus, hypothalamus, corpus callosum, and left anterior medial temporal white matter, in patients with NT1 disorder (12–14). In contrast, functional (fMRI) studies based on intercerebral connectivity have revealed impairment in the executive attention network (15) and abnormal functional connectivity (16, 17) in patients with NT1 disorder, and the presence of an alternative neural circuit that controls emotional responses to emotional challenges (18). Patients with NT1 disorder have a cortical neural network involved in the processing of rewards and emotions (19) with lower thresholds (20) or overactivation, associated with cataplexy (21, 22). Other studies have reported that alterations in brain connectivity and topology of some brain regions in patients with NT1 disorder are associated with lethargy, depression, and impulsive behavior (23).

However, the results of these studies are not entirely consistent, and very little work has been performed to assess the overall topological properties of whole-brain neural networks in patients with NT1 disorder. In addition, the above studies, especially those employing fMRI, were performed during the awake state. fMRI studies looking at functional brain network properties in patients with NT1 disorder in the sleep state, however, have not been reported.

The simultaneous acquisition of EEG and fMRI data provides a noninvasive method of investigating brain function during sleep (24) EEG data can be used to determine the stages of sleep, while fMRI analysis data can provide insight into the neural activity at different stages (25).

The aim of this study was to analyze the characteristic changes in brain functional connectivity during sleep, in patients with NT1 disorder, using noninvasive simultaneous EEG, fMRI and its relationship with neurobehavioral abnormalities.

## Materials and Methods

### Objectives

This study recruited 36 right-handed patients (male: female ratio, 21:15; age, 10.0–27.0 years) with NT1 disorder diagnosed according to the International Classification of Sleep Disorders (ICSD)-3 (26), from the Sleep Medicine Center of Shengjing Hospital, China Medical University. Another 33 right-handed healthy controls (male: female ratio, 19:14; age, 11.7–28.6 years) were recruited from the community. We excluded 19 participants: 4 patients with NT1 disorder and 3 volunteers withdrew from the PSG examination, and 7 patients and 5 volunteers failed to fall asleep, were unable to enter the N2 phase of sleep, or had excessive head motion resulted in failure to obtain fMRI data containing the complete sleep stage (including stage N1 and stage N2). Therefore, we analyzed data from 25 patients with NT1 (age 22.4 ± 6.9 years, 64% male) and 25 healthy controls (age 22.5 ± 3.8 years, 60% male). This study was approved by the ethics committee of Shengjing Hospital, China Medical University Medical College. In accordance with the Declaration of Helsinki, all participants had signed an informed consent form to participate in the experiment prior to the study.

Admission criteria for patients with NT1 disorder were as follows. According to the International Classification of Sleep Disorders for Episodic Sleep Disorders, a sleep specialist delivered a diagnosis based on 3 months of excessive daytime sleep and a clear clinical history of cataplexy. The final diagnosis was made by nocturnal PSG and the Multiple Sleep Latency Test (MSLT); the severity of EDS was measured using the ESS in patients with episodic sleeping disorder and healthy controls. In the ESS measure, participants were asked to describe, on a scale from 0 to 3, how likely they were to fall asleep in eight different situations, and if the total score was ≥ 10, a diagnosis of excessive daytime sleepiness was made (26).

Exclusion criteria for patients with NT1 disorder and healthy volunteers were as follows: subjects with other sleep disorders; current or previous severe physical or neurological disorders; routine MRI scan findings of brain abnormalities (tumors, hemorrhages, infarct foci); history of severe psychiatric or neurological disorders in the immediate family; current or previous psychiatric disorders such as depression, anxiety, or substance abuse; congenital genetic disorders; and presence of contraindications to MRI examination.

All subjects including patients with NT1 disorder and healthy volunteers, underwent a comprehensive neurological examination prior to the MRI scan to rule out peripheral and central nervous system disorders. The full procedure of the experiment, its purpose, clinical significance and precautions were explained to each subject before the start of scanning, and all patients with NT1 disorder and healthy volunteers who participated in this study signed an informed consent form.

### Epworth Sleepiness Score (ESS)

The ESS was used to measure the degree of daytime sleepiness in patients with NT1 disorder and healthy controls. In the ESS measure, participants were asked to describe, on a scale from 0 to 3, how likely they were to fall asleep in eight different situations, and if the total score was ≥ 10, a diagnosis of excessive daytime sleepiness was made (26).

### Polysomnography (PSG) and Assessment of Excessive Daytime Sleepiness

Prior to study participation, all patients were asked to abstain from drinking coffee or alcoholic beverages for one day. All night polysomnograms were recorded on a respiratory electronic series physiological monitoring system (Alice 6, Philips, Murrysville, FL, USA) the day before the MRI examination. PSG was recorded from 22:00 to 06:00, including EEG, electro-oculogram, electrocardiogram, etc. Standard EEG (frontal, central, occipital EEG: F4/M1, C4/M1, O2/M1, backup F3/M2, C3/M2, O1/M2), mandibular EMG (3 mandibular electrodes and EMG in the middle of the right tibialis anterior muscle), EOG (EOG located in the cornea and retina), and ECG were recorded according to the American Academy of Sleep Medicine guidelines. Oral and nasal airflow, snoring, chest and abdominal breathing, oxygen saturation and position, as well as total sleep time, sleep latency, sleep efficiency, arousal, and breathing events, were all recorded. According to the American Academy of Sleep Medicine Manual, a decrease in airflow of ≥ 90% and lasting for at least 10 s is defined as obstructive apnea and is associated with sustained respiratory effort; a decrease in airflow of ≥ 30% and lasting for at least 10 s is defined as hypopnea with 4% or higher oxygen desaturation (27). The apnea hypopnea index was calculated from the mean of the total number of apnea and hypopnea events experienced per hour of sleep. All patients completed a clinical MSLT the day after nocturnal PSG. With reference to the modifications approved by the American Academy of Sleep Medicine Task Force (28), naps were scheduled every 2 h starting 2 h after first awakening in the morning. If the patient did not sleep within 20 min, the NAP test was terminated, and the sleep latency was recorded for 20 min. If sleep occurred within 20 min, the onset time was defined as the time from lights out to the first sleep period (including stage 1). To assess the presence of rapid eye movement(REM)sleep, monitoring was continued for ≥ 15 min after sleep onset. If REM sleep was present, the latency of REM sleep was also recorded. The mean sleep latency and REM sleep latency of the five NAPs in the MSLT, were then calculated.

PSG data and EEG data were analyzed by an experienced technologist who had no knowledge of the clinical or demographic status of the participants. The results of the analysis were then verified by a physician. According to the American Academy of Sleep Medicine (AASM) criteria, participant's sleep was scored according to the staging of sleep, and related events, such as cortical arousal and wakefulness. The TST and % TST (NREM, including stages N1-N3 and REM sleep) for each sleep stage were calculated, along with the arousal index (A index, indicating observed sleep disruptions).

### Montreal Cognitive Assessment Beijing (MoCA-BJ)

All cognitive function tests were assessed using the MoCA-BJ (29). The test included cognitive domains such as visual-spatial and executive ability (5 points), naming (3 points), attention and computation (6 points), language (3 points), abstraction (2 points), delayed recall (5 points), and orientation (6 points), with a total score of 30 points and a duration of 10 min. A score of < 26 was considered cognitive impairment.

The Stanford Sleepiness Scale (SSS) (30) was used to asses sleepiness during the MoCA-BJ testing session. All participants filled in the scale before and after the testing session. The patients with NT1 had a mean score of 1.7 (SD = 0.9) before and 1.9 (SD = 1.1) after the test session. The healthy controls had a mean score of 1.1 (SD = 0.5) before and 1.4 (SD = 0.7) after the testing session.

### EEG-MRI Scan

Simultaneous EEG-fMRdeepI data were acquired within one week after multi-conductor EEG monitoring. All images were acquired using a Philips 3.0T superconducting MRI scanner (Philips Medical Systems, Best, Netherlands). The head scan coil was a 32-channel RF magnetic head coil and a net amplifier 300, with 32-channel electrode caps. The subject was placed in a comfortable supine position, flat on the MRI bed, wearing noise-reducing earplugs, and the subject's head was immobilized with foam padding, to reduce head movement. And the amount of impaired sleep in this study was within the same range as in previous studies (31). To exclude any obvious organic brain lesions, all patients with episodic sleeping sickness and healthy controls underwent a routine axial T2WI scan. The angular line was used as the baseline for the scan, and an experienced radiologist read the films on site at the end of the scan. The patient was informed of the scan time and instructed not to resist sleep during the entire EEG-fMRI scan. The subject was asked to keep the head as still as possible during the scan. When the subject signals showed that they could no longer sleep in the scanner, the scan was terminated.

The fMRI data were acquired using a gradient-echo plane echo sequence, with time repetition (TR) = 2 s, time echo (TE) = 30 ms, spatial resolution = 3.5 × 3.5 × 4 mm^3^, matrix size = 64 × 64, Field of View (FOV) = 220 × 220 mm, flip angle (FA) = 90°, sensitivity encoding factor (SEF) = 2, number of layers scanned. (number of slices) = 31, and layer thickness = 0 mm. The number of consecutive volume runs was collected 1,200 times, and an additional virtual scan (8 s in duration) was applied at the beginning of the fMRI scan, to stabilize the magnetic field and eliminate chemical displacement artifacts. Thus, the duration of the fMRI scan for each segment was 40 min and 8 s.

EEG data acquisition was synchronized with the MRI scanner clock. Thirty MRI-compatible scalp electrodes were placed in reference to the International 10–20 system; additional channels were used for ECG signal recording. The impedance of the electrodes was kept below 20 kΩ during signal acquisition. The sampling rate of the EEG data was 10 kHz. The frequency range of the hardware filter was 0.016 to 250 Hz.

### EEG Pretreatment and Sleep Staging

Net Stations 5.4 package was used to remove gradient and pulse artifacts from the EEG data. Pre-processed EEG data were sleep staged according to AASM criteria without overlap, with each epoch lasting 30 consecutive seconds, and then compared to a standard sleep montage by a qualified neurophysiologist.

### Functional Magnetic Resonance Imaging Preprocessing

The fMRI data were pre-processed on MATLAB version R2016a platform using the SPM12 package (Wellcome Trust Center for Neuroimaging at UCL, London, UK). The fMRI data from the different layers were first temporally corrected. The images were then re-aligned to the first layer of images for head motion correction and to estimate the head motion parameters (translation and rotation) for each direction. The resulting image space was then normalized to the Montreal Neurological Institute (MNI) labeled space with a resampled voxel of 3 × 3 × 3 mm. The half-height full width (FWHM) of the Gaussian kernel was set to 6 mm, and the normalized images were spatially smoothed; the spatially smoothed images were time-filtered (0.01-0.15 Hz). Finally, head movement parameters, ventricular signals, and brain white matter signals were regressed using the Data Processing and Analysis of Brain Imaging (DPABI) software package (32) (http://rfmri.org/dpabi). The pre-processed data were then divided into epochs lasting 30 consecutive seconds. Each epoch was assigned a particular sleep stage determined by simultaneously acquired EEG data. The minimum acceptable length of fMRI data for each sleep stage with a subject was 8 epochs (4 min).

### Brain Network Structure and Graph Theory Analysis

**Figure 1:**
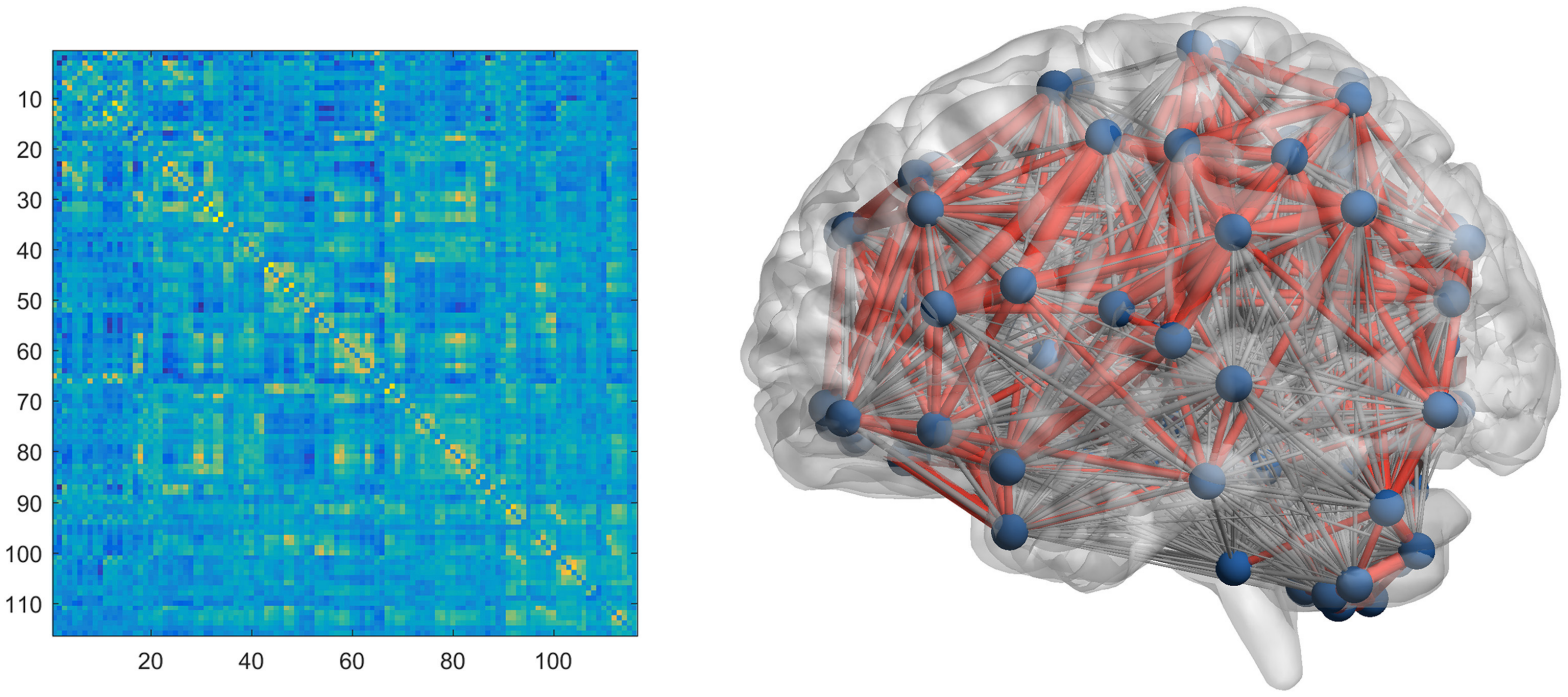
Construction of brain network in the participants.

A graph-theoretical network analysis toolbox, Gretna, was used to target the completed preprocessed fMRI data in order to construct functional brain networks in each for all epochs (33). To this end, the entire brain was divided into 90 cortical and subcortical regions based on automated anatomical label (AAL) markers (34), and the average time series of the 90 regions was extracted. A Pearson's correlation coefficient for each pair of regions was calculated for the average time series of all 90 regions, and the data were transformed into z-values, considered to have a normal distribution using Fisher's z-transform. A positively binary vector less connected generic network was then constructed based on the chosen threshold range of the correlation matrix. After conversion, the z-scored correlation coefficients assigned to the same sleep stage were averaged for each participant (Figure 1).

Graph theory analysis was used to assess the topological and organizational properties of the entire brain. Topological measures can be divided into global efficiency (Eglob) and local efficiency (Eloc) (35) and small-world network parameters (36) (Supplement 1). Sparsity was used as a correlation measure for the correlation coefficient threshold range, defined as the number of available edges in the graph divided by the maximum number of possible edges. According to previous research (35), we set both the sparsity and Pearson correlation thresholds of the function network in the range of 0.05 to 0.5 (in 0.05 steps) to obtain more efficient ubiquitous networks, when compared to random networks with the lowest number of artificial edges.

### Data Analysis

This study used IBM SPSS software (version 17, IBM Inc., Armonk, NY, USA) to analyze demographic, cognitive data, brain function network data, and PSG data. The Kolmogorov-Smirnov test was used to test the hypothesis of normality for continuous variables. Descriptive statistics were firstly performed. Continuous data with a normal distribution were expressed as mean ± standard deviation (SD), and non-normally distributed data were expressed as the median of the interquartile range. These data include age, and questionnaire score. Comparisons were made between the patient with NT1 disorder group and the healthy control group, using *t*-tests or Mann Whitney *U*-tests based on the normality and homoscedasticity of these variables. A χ2 or Fisher's exact test was taken to compare sex ratios between the different groups. The demographic characteristics of the subjects who dropped out were also compared with those of the subjects who remained. The *t*-test or Mann-Whitney *U*-test was also used to compare PSG data and topological parameters of the brain functional network during sleep between the two groups. The Holm-Bonferroni method was used to correct for multiple comparisons. Statistical significance for all the above statistical analyses required a two-tailed test with a level < 0.05.

A Spearman's correlation analysis was used to explore the relationship between MoCA and brain network parameters in patients with NT1 disorders. *P* < 0.05 was defined as a significant difference.

## Results

This study contains demographic, PSG, MoCA-BJ scale, and imaging data for 25 patients with NT1 disorder and 25 healthy volunteers (50 participants in total).

Table 1 summarizes the demographics of the participants. The two groups were matched for age and sex. In the healthy control group: age 22.5 ± 3.8 years, 60% male; and in the NT1 disorder patient group: age 22.4 ± 6.9 years, 64% male. The MoCA-BJ score was significantly lower in the patient with NT1 disorder group than in the control group (*P* < 0.001).

**Table 1:** Demographics data of study participants.

	**Patients (*n* = 25)**	**Healthy controls (*n* = 25)**	***Chi-square*, *t* or *Z* value**	***P* value**
Age	22.4 (6.9)	22.5(3.8)	0.063	0.95
Sex (% male)	16 (64%)	15 (60%)	0.085	0.77
MoCA-BJ scores	25 (25–26)	30 (0–0)	25	<0.0001^#, *^
Disease duration (years)	7.1 (3.9)	-		

*Data were presented as median (IQR), frequency (%), or mean (SD)*.

# *P value were corrected using Holm-Bonferroni method*.

* *P < 0.05*.

### PSG Data

**Figure 2:**
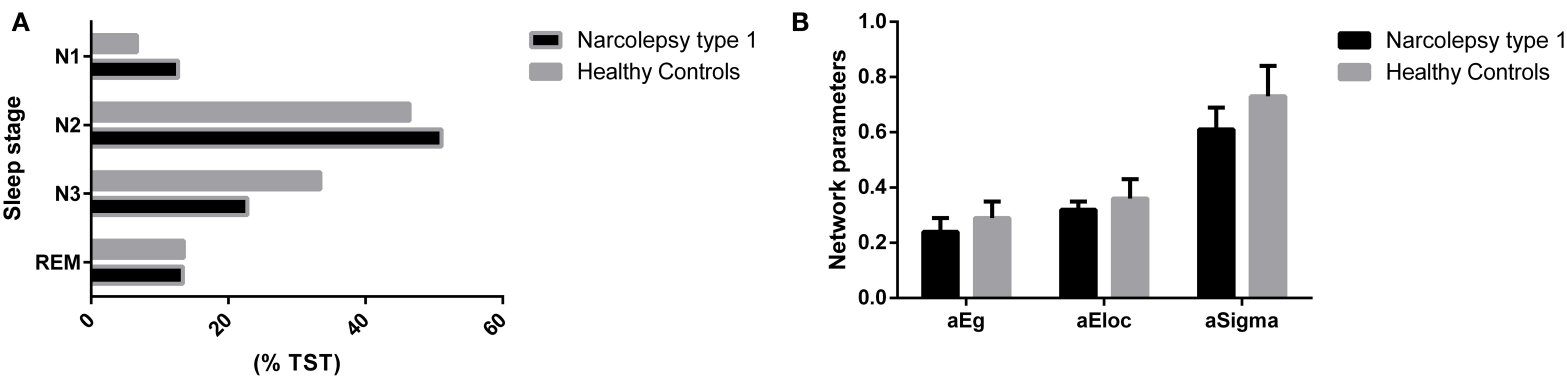
Comparisons of %TST and cerebral topological parameters between groups. **(A)** Compared with the control group, patients with NT1 disorder had significantly higher percentage of N1 and N2 during TST (*t* = 8.479, *P*_*corrected*_ < 0.0001; *t* = 3.426, *P*_*corrected*_ < 0.001) and significantly lower %TST during N3 (*t* = 4.655, *P*_*corrected*_ < 0.0001). **(B)** Compared with the control group, patients with NT1 disorder had significantly lower global efficiency and small-world attributes (Bonferroni correction, *P* < 0.05).

Patients with NT1 disorder had significantly higher percentage of N1 and N2 during TST (*t* = 8.479, *P*_corrected_ < 0.0001; *t* = 3.426, *P*_corrected_ < 0.001) and significantly lower %TST during N3 (*t* = 4.655, *P*_corrected_ < 0.0001), when compared to controls. The A index was significantly higher in patients with NT1 disorder than in the control group (*t* = 10.630, *P*_corrected_ < 0.0001), but the TST was not significantly different in patients with NT1 disorder, when compared to the control group (*P* > 0.05) (Table 2, Figure 2).

**Table 2:** PSG data in NT1 participants and healthy controls.

	**Patients (*n* = 25)**	**Control (*n* = 25)**	***Chi-square, t* or *Z* value**	***P* value**
TST	7.0 (0.8)	7.2 (0.9)	0.831	0.41
Sleep stage (% TST)				
N1	12.7 (2.6)	6.7(2.4)	8.479	<0.0001^#, *^
N2	51.1 (4.8)	46.4 (4.9)	3.426	0.001
N3	22.8(8.2)	33.4 (7.9)	4.655	<0.0001^#, *^
REM	13.4 (7.3)	13.5 (6.1)	0.053	0.96^#^
A index	21.5 (7.9)	4.0(2.3)	10.630	<0.0001^#, *^

*Data were presented as median (IQR), frequency (%), or mean (SD)*.

# *P value were corrected using Holm-Bonferroni method*.

* *P < 0.05*.

### Brain Network Analysis

**Figure 3:**
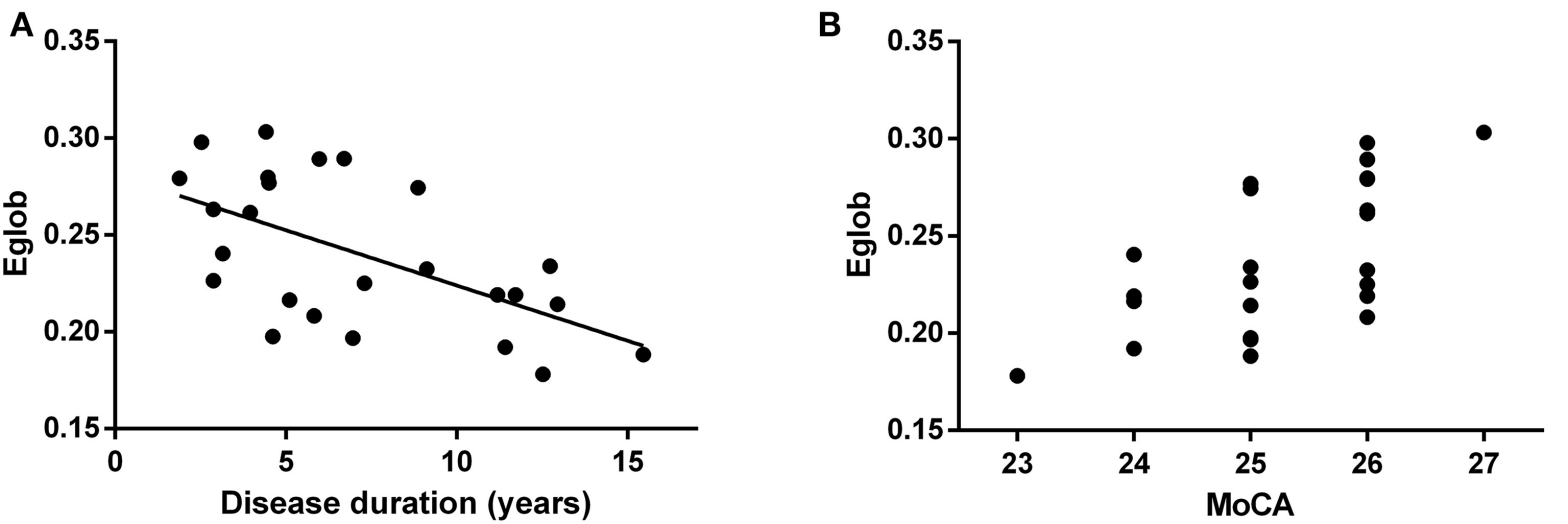
Relationship between the global efficiency of the whole brain functional network, and years of onset, and MoCA score in NT1 participants. **(A)** There was a significant correlation between the global efficiency of the whole brain functional network, in patients with NT1 disorder, and Disease duration in the stage N2 sleep. **(B)** There was a significant correlation between the global efficiency of the whole brain functional network, in patients with NT1 disorder, and MoCA score in the stage N2 sleep.

Patients with NT1 disorder had significantly lower global efficiency and small-world attributes when compared to healthy controls (Bonferroni correction, *P* < 0.05, Table 3). There was a significant correlation between the global efficiency of the whole brain functional network in patients with NT1 disorder, years of onset, and MoCA-BJ score in the stage N2 sleep (*r* = −0.589, *P* = 0.002; *r* = 0.632, *P* < 0.001, respectively) (Figure 3).

**Table 3:** Brain functional network parameters in NT1 participants and healthy controls.

	**Patients (*n* = 25)**	**Healthy controls (*n* = 25)**	***Chi-square, t* or *Z* value**	***P* value**
aEg	0.24 (0.05)	0.29 (0.06)	3.201	<0.007^#, *^
aEloc	0.32(0.03)	0.36(0.07)	2.626	<0.015^#, *^
aSigma	0.61(0.08)	0.73 (0.11)	4.927	<0.001^#, *^

*Data were presented as median (IQR), frequency (%), or mean (SD)*.

# *P value were corrected using Holm-Bonferroni method*.

* *P < 0.05*.

## Discussion

In this study, we found that patients with NT1 disorder have a higher proportion of stage N1 and stage N2 during all night PSG monitoring, which is consistent with previous studies (37). Since it was difficult for participants to reach the stage N3 during MRI scanning due to the noisy environment and only N2 period data of fMRI scanning in this study were available to analyze, this study only analyzed fMRI data from the stage N2 sleep.

Our study found that the global efficiency and small-world properties of the whole brain functional network in patients with NT1 disorder were lower than those in normal controls during stage N2 sleep. Our data suggested that the global efficiency was proportional to the length of onset and correlated with the severity of cognitive impairment. Since the global efficiency of brain networks is mainly influenced by long-distance connections within the network, we speculated that the reduced topological efficiency of brain networks during sleep, may be primarily due to abnormalities in long-distance functional connections. These abnormalities in the topological efficiency of brain networks may be related to the connection anomalies between several brain networks previously found in resting state studies, including altered connectivity between the executive and salience networks (16), reduced inter-node connectivity in the default mode network (38), and reconfiguration of the salience and default mode networks (23). Given previous studies have demonstrated that the brain sensory-motor networks in the resting state, such as the somatomotor network, visual network, auditory network, default mode network, dorsal attention network, abdominal attention network, linguistic network, frontal parietal network, salient network, always exhibited constant neurological activity (39). Thus, the inefficiency of the functional brain networks in the sleep state of NT1 disorder patients is likely to lead to a decrease in information processing ability in the sleep state, which in turn extends to the waking state leading to cognitive impairment.

Although this EEG-fMRI study provides preliminary evidence of abnormalities in brain functional networks in patients with NT1 disorder during sleep, this study has several limitations. First, the number of patients with NT1 disorder was limited. Second, the age distribution of the patients was large. Third, this study only analyzed fMRI data from the stage N2 sleep.

In summary, we found that patients with NT1 disorder have reduced whole-brain functional network efficiency during stage N2 sleep, and that this reduced whole-brain functional network efficiency may be related to their cognitive impairment.

## Data Availability Statement

The raw data supporting the conclusions of this article will be made available by the authors, without undue reservation.

## Ethics Statement

The studies involving human participants were reviewed and approved by Ethical Committee of Shengjing Hospital of China Medical University. The patients/participants provided their written informed consent to participate in this study.

## Author Contributions

XZ: data curation and writing-original draft preparation. KN: data curation and software. HT: visualization and investigation. YL: writing-reviewing and editing. YZ: validation. BY: conceptualization and methodology. LX: conceptualization, validation, and supervision. All authors contributed to the article and approved the submitted version.

## Conflict of Interest

The authors declare that the research was conducted in the absence of any commercial or financial relationships that could be construed as a potential conflict of interest.
