# Supplementary material for: Abnormal Brain Network Topology During Non–rapid Eye Movement Sleep and Its Correlation With Cognitive Behavioral Abnormalities in Narcolepsy Type 1
Source: Front Neurol. 2021 Jan 11;11:617827. doi: 10.3389/fneur.2020.617827 (PMC7829333; doi:10.3389/fneur.2020.617827)
Supplement: Supplementary file 1 [file Data_Sheet_1.docx]

**Supplement 1**

**Network Properties.**

The **path length** between any pair of nodes (e.g., node i and node j) is defined as the reciprocal of the edge weight, 1/wij. The shortest path length, Lij, is defined as the length of the shortest path for node i and node j. The shortest path length of a network is computed as follows:


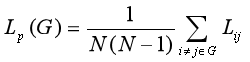
 Eq(1)

where N is the number of nodes in the network. The Lp of a network quantifies its ability for the parallel propagation of information.

The **global efficiency** of *G* measures the global efficiency of the parallel information transfer in the network (Latora and Marchiori, 2001), which can be computed as:


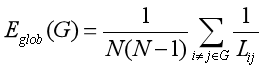
 Eq(2)

where *L_ij_* is the shortest path length between node i and node j in G.

The **local efficiency** of *G* reveals how fault tolerant the network is and reveals how efficient the communication among the first neighbors of the node i is when node i is removed. The local efficiency of a graph is defined as:


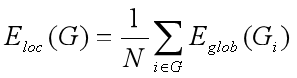
 Eq(3)

where *G_i_* denotes the subgraph composed of the nearest neighbors of node i.

To examine the small-world properties, the clustering coefficient, *Cp*, and shortest path length, Lp, of the brain networks were compared with those of random networks. In this study, 100 matched random networks were generated, which had the same number of nodes, edges, and degree distributions as the real networks. The weight of each edge during the randomization procedure was retained to preserve the weight distribution.

After random networks generation，the normalized shortest path length (lambda),
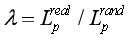
, and the normalized clustering coefficient (gamma),
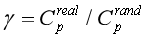
, where
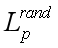
 and
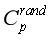
 are the mean shortest path length and the mean clustering coefficient of the 100 matched random networks, respectively were calculated respectively. The two measurements were summarized in a simple quantitative metric termed small-worldness,


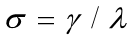
 Eq(4)

which is typically greater than 1 for small-world networks.
